# A Longitudinal Investigation of the Relationship Between Trauma-Related Cognitive Processes and Internalising and Externalising Psychopathology in Young People in Out-of-Home Care

**DOI:** 10.1007/s10802-022-01005-0

**Published:** 2022-12-16

**Authors:** Rebecca S Davis, Sarah L Halligan, Richard Meiser-Stedman, Elizabeth Elliott, Georgina Ward, Rachel M Hiller

**Affiliations:** 1 Department of Psychology, University of Bath, Bath, UK; 2 Department of Psychiatry and Mental Health, University of Cape Town, Cape Town, South Africa; 3 Department of Clinical Psychology and Psychological Therapies, University of East Anglia, Norwich, UK; 4 Division of Psychology and Language Sciences, University College London, London, UK; 5 Anna Freud National Centre for Children and Families, London, UK

**Keywords:** Complex Trauma, Maltreatment, Foster Care, Internalising, Externalising, PTSD, Cognitive Processes

## Abstract

Young people in out-of-home care are at increased risk of developing a range of posttrauma mental health difficulties, including PTSD, but more commonly anxiety, depression and externalising symptoms. Cognitive models of PTSD indicate that trauma-related maladaptive appraisals, coping strategies and trauma memory qualities are key processes in the development and maintenance of PTSD, yet there has been limited investigation of the potential role of these processes in broader posttrauma psychopathology, particularly in young people who have been exposed to complex, rather than acute, trauma. We recruited 120 10–18 years olds in out-of-home care, and their caregivers, who completed assessments at two time points: baseline and 12-month follow-up. Young people completed self-report measures of trauma-related maladaptive appraisals, coping strategies and trauma-memory qualities, as well as reporting on PTSD, anxiety, depression and externalising symptoms. Carers also reported on internalising and externalising symptoms. We found that all three cognitive processes were associated with baseline self-reported internalising symptoms, with maladaptive appraisals most robustly associated with both anxiety and depression. Changes in all three processes over 12-months predicted a change in self-reported internalising and externalising symptoms, with maladaptive appraisals and coping predicting anxiety symptoms, and coping uniquely predicting depression and externalising symptoms. Effects remained after controlling for co-occurring PTSD symptoms. Findings were not replicated when using carer-reported symptoms. These findings suggest that existing cognitive models of PTSD may also usefully explain broader posttrauma depression, anxiety and externalising symptoms in young people who have experienced maltreatment and live in out-of-home care. Clinical implications are discussed.

Exposure to a traumatic event in childhood is a key risk factor for the development of later mental health difficulties (Carr et al., 2013). Exposure to trauma which is considered complex (i.e., experiences which are often prolonged, repeated, and interpersonal in nature, such as childhood maltreatment) confers an even greater risk of developing severe mental health difficulties (Lewis et al., 2021). One group of young people who often have a history of complex trauma exposure are children and teens in out-of-home care (i.e., under the care of the child welfare/social care system). Most young people in out-of-home care have experienced maltreatment, alongside many other adversities, such as parental mental health difficulties, parental drug and alcohol misuse, and poverty (Department for Education [DfE], 2021; Simkiss et al., 2012). Once in care, many continue to face ongoing instability, including frequent changes in caregiver and separation from siblings (Ashley & Roth, 2015; DfE, 2021). Rates of further exploitation can also be high for some groups, particularly teens placed in residential or semi-independent living arrangements (Parliament, House of Lords, 2012). Such experiences reflect an accumulation of risk, trauma and adversity, which is often considered particularly complex in nature. While some young people in care do experience stability and thrive, it is also the case that many experience high rates of psychological distress, with a meta-analytic review estimating that around 50% meet criteria for at least one psychiatric disorder (Bronsard et al., 2016). Although the high rates of mental health difficulties experienced by this group are well-known, there has been less attention on psychological processes which may lead to the development and maintenance of these difficulties. Yet, such information is essential for informing intervention development.

Much of the attention in the child trauma field has focused on posttraumatic stress disorder (PTSD) – a trauma-specific mental health outcome. Cognitive models of PTSD have provided a useful framework for understanding why PTSD might develop after a young person is exposed to trauma. Ehlers and Clark’s (2000) cognitive model highlights three key cognitive processes which serve to maintain a sense of current threat. One such process is maladaptive appraisals or cognitions. If a traumatic event and its sequalae are appraised in a highly negative manner, this can lead to a global sense of external and internal threat which extends beyond the event itself, for example, believing that the world is a dangerous place and that they are no longer a capable person (de Haan et al., 2020; Ehlers & Clark, 2000). The quality of the trauma memory is also considered a key factor, both in terms of memory characteristics and the overall narrative. Trauma memories tend to be characterised by a higher level of sensory information and less semantic or contextual information, meaning they are more fragmented and disorganised (Salmond et al., 2011). The nature of how these memories are encoded and stored means that they are susceptible to being triggered involuntarily by environmental or internal cues and experienced as ‘flashbacks’, whereby a person feels as though they are reexperiencing the traumatic event, serving to maintain an ongoing sense of threat. Lastly, attempts to manage an ongoing sense of threat can lead to maladaptive cognitive and behavioural coping strategies, such as thought suppression and cognitive avoidance (e.g., trying to avoid thoughts about the trauma). In the short term these strategies may serve to reduce feelings of distress, however in the longer-term they can prevent changes in how information is appraised or how trauma memories are processed, leading to a maintenance of PTSD symptoms (Hiller et al., 2021; Trickey et al., 2012).

There is relatively robust evidence regarding the role of negative appraisals (Gomez de La Cuesta et al., 2019; Mitchell et al., 2017) and maladaptive coping strategies (Trickey et al., 2012) in the development and maintenance of PTSD in young people exposed to acute (one-off) trauma. The evidence regarding trauma memory qualities and PTSD is less clear. While some studies have found that traumatic stress reactions are associated with memories which are more sensory and disorganised in nature (Kenardy et al., 2007; Salmond et al., 2011), others have reported memories which are more cohesive and less sensory (O’Kearney et al., 2007). More recently there is evidence that self-reported trauma memory characteristics (relating to sensory qualities and disorganisation) may be a better predictor of PTSD symptoms than the nature of the trauma memory narrative itself (McGuire et al., 2021; McKinnon et al., 2017). For young people exposed to more complex trauma, including young people in care, there is emerging evidence of the relevance of these cognitive processes. A recent systematic review found general support for the role of all three processes in the development and maintenance of PTSD in young people who have experienced maltreatment, although the lack of research into the role of trauma memory qualities limited the support for this process (Wiseman et al., 2019). For young people in care specifically, moderate to strong associations have been found for the three combined cognitive processes, with maladaptive appraisals the most robust driver of both concurrent and longer-term PTSD symptoms (Hiller et al., 2021).

While there is good evidence regarding key cognitive mechanisms which drive PTSD in trauma exposed young people, PTSD is not the most common mental health outcome in young people exposed to trauma. The prevalence rates of other internalising difficulties, such as depression, as well as externalising difficulties (e.g., conduct disorder), are often higher than rates of PTSD (Bronsard et al., 2016; Lewis et al., 2019). Additionally, those young people who go on to develop PTSD after experiencing trauma are significantly more likely to develop comorbid mental health difficulties, than those who do not develop PTSD (Lewis et al., 2019). Given the high rates of other mental health difficulties in this population, and comorbidities alongside PTSD being commonplace, it is useful to explore the potential transdiagnostic role of trauma-related cognitive processes. Exploring the role of trauma-related processes that may underlie a range of mental health outcomes has important implications both for our understanding of the applicability of existing cognitive models of PTSD for other psychopathology, as well as adding to the limited evidence-base of processes that may maintain broader difficulties in those in out-of-home care, and thus form potential treatment targets.

For young people who have experienced acute trauma, there is evidence indicating that maladaptive trauma-related appraisals are cross-sectionally associated with depression and generalised anxiety disorder (Liu & Chen, 2015). Trauma memory qualities and maladaptive coping styles, in addition to negative appraisals, have also been found to be associated with self-reported anxiety and depression symptoms six-months post acute trauma, with negative appraisals and maladaptive coping uniquely predicting internalising symptoms (Hiller et al., 2019). Within the acute trauma literature, studies have reported contradictory findings regarding the role of trauma-related cognitive processes and externalising difficulties. Liu and Chen (2015) found a positive cross-sectional association between negative appraisals and caregiver-reported externalising symptoms, however Hiller et al. (2019) found that appraisals did not predict later caregiver-reported externalising difficulties.

For young people exposed to more complex trauma, such as maltreatment, there is some evidence highlighting significant cross-sectional associations between negative appraisals and internalising difficulties (de Haan et al., 2017; Leeson & Nixon, 2011). There is also evidence suggesting that the association between negative appraisals and depression symptoms may be greater for young people who have been exposed to interpersonal trauma, than those exposed to accidental trauma (de Haan et al., 2019). As with the acute trauma literature, for young people exposed to complex trauma, the association between trauma-related cognitive processes and externalising symptoms, both self- and caregiver-reported, is less robust (de Haan et al., 2017; Leeson & Nixon, 2011). However, there is evidence to indicate that posttrauma negative appraisals and maladaptive coping (e.g., cognitive avoidance) play a role in mediating the relationship between maltreatment and related risk outcomes, such as substance abuse (e.g., Allwood et al., 2014; Hogarth et al., 2019; Shin et al., 2020). Given the limited evidence, further research with young people exposed to complex trauma is required to understand the role of existing cognitive models of PTSD in the development of other mental health difficulties commonly experienced by this group.

The aim of the current study was to investigate the contribution of trauma-related cognitive processes to broader (non-PTSD) internalising and externalising difficulties in a sample of young people in out-of-home care (Hiller et al., 2021). Using a longitudinal design, we explored the role of three core cognitive processes outlined in the Ehlers and Clark’s (2000) cognitive model of PTSD: negative appraisals, trauma memory quality, and maladaptive cognitive coping. We explored the concurrent and longitudinal associations between these processes and self-reported depression, anxiety, and externalising symptoms, as well as carer-reported child internalising and externalising difficulties. Given the significant overlap between symptoms of PTSD and internalising and externalising symptoms (Lewis et al., 2019), and that these cognitive processes are significantly associated with symptoms of PTSD (Hiller et al., 2021), we also explored whether the three processes made a unique contribution to broader psychopathology, after controlling for PTSD symptoms.

## Method

### Participants

Participants were 120 young people in care aged 10–18 years, and their primary carer, recruited through social workers from three local authorities in England (full details of recruitment and ethical procedures, and the sample are reported in Hiller et al. (2021)). The majority of participants were living with a nonrelated foster carer (86%), with 9% in a kinship placement and 5% in a residential or semi-independent placement. Exclusion criteria were the presence of a severe learning or neurodevelopmental difficulty which precluded mainstream schooling, significant current suicidal ideation or psychosis, or a level of English which prevented the young person from completing the questionnaires.

### Procedure

Ethical Approval was obtained from the University of Bath and Social-Care Research Ethics Committees (Ref: 16/1EC08/0025), as well as participating Local Authorities. Safeguarding and risk escalation procedures were in place, and referrals to mental health services were made, when necessary and available. Social workers provided informed consent for 242 young people to be contacted about the study and, of these, 120 young people provided informed assent (or consent if they were over the age of 16 years). Of the 50.5% who declined participation, the main reasons were participants not being contactable or being too busy to take part. There were no significant demographic differences identified between those who took part and those who did not (see Hiller et al., 2021). Questionnaire packs were completed either during a home visit, online or via the post, at a baseline assessment and a 12-month follow-up. Of the original sample, 80% were retained at the 12-month follow-up. Between those who participated at the 12-month follow up and those who did not, there were no significant differences with regards to initial mental health symptoms or sex of the child. There was a non-significant trend of non-completers being slightly older (*p* = .052).

### Measures

#### Maltreatment History

To assess maltreatment severity, the young person’s social worker provided information regarding maltreatment history relating to physical abuse, sexual abuse, emotional abuse and domestic violence using Kaufman’s 5-point Likert scale (Kaufman et al., 1994), answering on a scale from 0 (*young person did not experience that type of abuse)* to 4 (*most severe*). Social workers also reported whether or not the young person had experienced 10 possible types of neglect. The four abuse subscales were totalled to provide a total abuse severity score (range 0–16), alongside a neglect severity score (range 0–10), with higher scores reflecting greater severity.

#### Maladaptive Trauma Appraisals

The Children’s Post Trauma Cognitions Inventory (CPTCI; Meiser-Stedman et al., 2009), a 25-item self-report measure, was used to assess young people’s negative trauma appraisals. This measure assesses appraisals relating to being a fragile person in a scary world (e.g., “*I don’t trust people”*) and a sense of enduring and distressing change since their traumatic experiences (e.g., “*bad things will always happen*”). Each item is rated from 1 (*Don’t agree at all*) to 4 (*Agree a lot*), with total scores ranging from 25 to 100 (α = 0.96), and higher scores reflecting more maladaptive appraisals.

#### Posttrauma Coping

The Child Posttrauma Coping Questionnaire (CPCQ; Hiller et al., 2018), an 11-item self-report measure, was used to measure rumination and cognitive avoidance. Items assess whether the young person is ruminating about a traumatic event (e.g., “*I keep wondering again and again why it happened to me”*) and the extent to which they are engaging in cognitive avoidance through thought suppression (e.g., “*I’ve tried to stop myself having any thoughts about the frightening event*”). Each item is rated from 0 (*Not at all or only one time*) to 3 (*A lot of the time*) with total scores ranging between 0 and 33 (α = 0.91) and higher scores indicating increased maladaptive coping.

#### Trauma Memory Qualities

The Adapted Child Trauma Memory Quality Questionnaire (ACTMQQ; Hiller et al., 2019), an 18-item self-report measure, was used to assess the nature of young people’s trauma memories. Items measure the sensory quality of the trauma memories (e.g., *“My memories of the frightening event are mostly pictures or images”*), their sense of “nowness” (e.g., “*When I remember the events, I feel like it is happening right now”*), as well as how coherent the memories are (*“My memories of the events are muddled”*). Each item is rated from 1 (*Disagree a lot*) to 4 (*Agree a lot*), yielding a total score range of 18–72, with higher scores indicating memories which are more disorganised and contain more sensory information. This adapted scale showed good internal consistency (α = 0.88) and has established validity against child PTSD symptoms measures (Hiller et al., 2019).

#### Anxiety and Depression Symptoms

The Revised Child Anxiety and Depression Scale (RCADS; Ebesutani et al., 2017), a 25-item self-report questionnaire, was used to assess anxiety and depression symptoms. The measure contains 15-items measuring anxiety symptoms (total score range = 0–45; e.g., “*I worry that something bad will happen to me*”) and 10 questions measuring symptoms of depression (total score range = 0–30; e.g., “*I feel sad or empty*”). Each item is rated from 0 (*Never*) to 3 (*Always*) with higher scores indicating a higher level of anxiety or depression symptoms. Internal consistency was good at both timepoints (anxiety: α > 0.88, depression: α > 0.92).

#### Externalising Symptoms

The 10-item externalising subscale of the widely validated Strengths and Difficulties Questionnaire (SDQ; Goodman, 2001) was used to assess self-reported externalising symptoms. This subscale is made up of a 5-item conduct problems scale and a 5-item hyperactivity/attention scale. Higher scores are indicative of a higher level of externalising symptoms. Internal consistency over both timepoints was good (α > 0.80).

#### Carer-Reported Externalising and Internalising Symptoms

The Strengths and Difficulties Questionnaire (SDQ; Goodman, 2001) was also used to measure carer-reported child symptoms, utilising both the 10-item externalising subscale and 10-item internalising subscale. Internal consistency for each subscale over both timepoints was good (externalising: α > 0.85, internalising: α > 0.79). For young people in residential or semi-independent care, the SDQ was completed by their keyworker (a key professional who provides support for the young people in their placement).

#### PTSS

The Child and Adolescent Trauma Screen (CATS), a 20-item self-report questionnaire, was used to measure symptoms of PTSD. Each item is rated from 0 (*Never*) to 3 (*Almost always*) with total scores ranging from 0 to 60 and higher scores reflecting greater symptom severity. The CATS has been found to be a reliable and valid assessment of PTSD symptoms that corresponds with the DSM-5 (Sachser et al., 2017). The measure showed excellent internal consistency at both timepoints (α > 0.94).

### Data Analysis Plan

Analyses were conducted using SPSSv25. As all mental health outcomes were positively skewed, square root transformations were applied. Despite transformation, the majority of outcomes at both time points remained skewed and so associations were checked using non-parametric tests (Spearman’s rho), with any discrepancies noted (see Table 1). We conducted correlational analyses to establish if the three cognitive processes (negative appraisals, trauma memory qualities, coping strategies) were associated with mental health outcomes (self-reported depression, anxiety and externalising symptoms, and carer-reported externalising and internalising symptoms) at baseline, and at 12 months. Age, sex and maltreatment severity were tested as potential covariates.

**Table 1:** *Bivariate Correlation Matrix for Associations Between Study Variables*

Variable	1.	2.	3.	4.	5.	6.	7.	8.	9.	10.	11.	12.	13.
Baseline processes													
1. Appraisals													
2. Coping	0.71**												
3. Memory	0.61**	0.64**											
Baseline outcomes													
4. Anxiety	0.79**	0.67**	0.64**										
5. Depression	0.82**	0.62**	0.56**	0.80**									
6. Externalising	0.58**	0.39**	0.29**	0.41**	0.54**								
7. Carer-report externalising	0.24*	0.22*	0.14	0.21*^a^	0.23*	0.59**							
8. Carer-report internalising	0.34**	0.33**	0.23*	0.44**	0.46**	0.27**	0.58**						
12-month outcomes													
9. Anxiety	0.47**	0.35**	0.42**	0.57**	0.52**	0.34**	0.07	0.30**					
10. Depression	0.57**	0.41**	0.33**	0.59**	0.67**	0.36**	0.08	0.36**	0.78**				
11. Externalising	0.36**	.19^a^	0.23*^a^	0.29**	0.41**	0.61**	0.44**	0.31**	0.51**	0.55**			
12. Carer-report externalising	0.10	0.08	0.08	0.17	0.04	0.46**	0.73**	0.33**	0.21	0.17	0.56**		
13. Carer-report internalising	0.37**	0.38**	0.17	0.46**	0.34**	.10^a^	0.47**	0.77**	0.36**	0.38**	0.39**	0.52**	
*M (SD)*	41.00 (17.19)	10.57 (8.95)	33.36 (10.47)	7.03 (7.44)	5.25 (6.49)	7.39 (4.33)	7.83 (5.03)	6.16 (4.24)	5.89 (7.04)	4.77 (5.96)	6.52 (4.15)	7.28 (4.61)	5.69 (4.10)

*Note*. Anxiety, depression and externalising symptoms were measured via self-report

* *p* < .050, ** *p* < .010

^a^ – Using Spearman’s rho, baseline carer-reported externalising symptoms were no longer associated with baseline anxiety symptoms; 12-month self-reported externalising was no longer associated with trauma memory; 12-month self-reported externalising was associated with coping; 12-month carer-reported internalising was associated with baseline self-reported externalising. There were no other discrepancies between parametric and non-parametric tests.

To investigate the extent to which the combined baseline cognitive processes predicted symptom severity at 12-months, separate hierarchical regressions were run for depression, anxiety, externalising symptoms and carer-reported externalising and internalising symptoms, while controlling for covariates. We then re-ran each model, controlling for concurrent PTSS, to test whether any associations were secondary to PTSS.

To explore longitudinal associations between changes in cognitive processes and symptom change, residual change scores were calculated for each cognitive process and outcome measure (i.e., the residual score at follow up once the baseline score for that variable was accounted for in a regression model). Separate hierarchical linear regressions were run for each mental health outcome, with change scores entered in a single step, with symptoms at 12-months as the dependent variable, while controlling for baseline symptoms and covariates. Again, these regression models were re-run while also controlling for changes in PTSS over time (i.e., PTSS residual change scores), in order to ascertain whether differences in the cognitive processes over time continued to predict symptom change, beyond what could be explained by PTSS.

Sensitivity analyses were carried out in order to assess the robustness of our findings. Follow-up mental health data were missing for 27 to 30% of cases. To account for missing data, regressions were re-run following multiple imputation using 50 iterations and predictive mean matching. The pattern of results was largely the same and any discrepancies have been reported.

## Results

### Descriptives and Covariates

The average age of participants was 13.5 years (*SD* = 2.2) and just over half were female (53%). The average age at which they had entered care was 8.3 years (range 0–16 years). At baseline, the majority of young people were in a planned long-term placement (89%) but by 12-months, a quarter of the young people had moved placement (25%). Based upon social worker report, 97% of the sample had experienced at least one form of abuse and 96% had experienced neglect. Of these, 44% of young people had experienced physical abuse, 18% had experienced sexual abuse, 87% had experienced emotional abuse, and 77% had witnessed domestic violence.

Sex, age and maltreatment (abuse and neglect) severity were examined as potential covariates. Sex was significantly associated with self-reported anxiety (*rs* > 0.30, *p* < .002), depression (*rs* > 0.20, *p* < .041) and PTSD symptoms (*rs* > 0.27, *p* < .005) at both timepoints, with females having higher average symptom scores. Age was positively associated with depression at baseline (*r* = .22, *p* = .025) and negatively associated with carer-reported externalising symptoms at 12-months (*r* = − .33, *p* = .002). Total abuse severity was associated with carer-reported externalising symptoms at both time points (*rs* > 0.20, *p* < .039), and total neglect severity was associated with carer-reported externalising symptoms at 12-months (*r* = .25, *p* = .028). Neither were associated with any self-report outcomes. For consistency, age and sex were added as covariates in all regressions, and maltreatment (abuse and neglect) severity was included as a covariate in regressions involving carer-reported mental health outcomes.

There were no significant changes in cognitive processes or symptoms scores over the 12-months, apart from a reduction in maladaptive coping (*p* = .020).

### Anxiety

Each cognitive process was positively associated with self-reported anxiety symptoms at baseline (Table 1), and changes in each cognitive process were associated with changes in anxiety symptoms over 12-months (Table 2). Regression analysis, controlling for age and sex, showed that the combined cognitive processes accounted for 59% of variance in anxiety symptoms at baseline, with negative appraisals the sole unique predictor (β = 0.59, *p* < .001). When additionally controlling for baseline PTSS, the combined cognitive processes continued to predict anxiety symptoms (accounting for 12% of variance), and negative appraisals remained the only unique predictor (β = 0.48, *p* < .001) (Table 3). Of note, baseline PTSD symptoms were not uniquely associated with baseline anxiety symptoms (*p* = .157).

**Table 2:** *Bivariate Correlations Between Residual Change Scores*

	1.	2.	3.	4.	5.	6.	7.	8.
1. Appraisals								
2. Coping	0.72**							
3. Memory	0.73**	0.64**						
4. Anxiety	0.71**	0.74**	0.66**					
5. Depression	0.58**	0.62**	0.53**	0.65**				
6. Externalising	0.31**	0.48**	0.37**	0.48**	0.46**			
7. Carer-report externalising	0.07	0.29*	0.12	0.21	0.29*	0.48**		
8. Carer-report internalising	0.09	0.19	0.15	0.17	0.27*	0.34**	0.46**	
*M* difference score ^a^ (*SD*)	-1.65 (16.09)	-2.26 (10.21)	-1.59 (10.34)	-0.86 (7.15)	-0.29 (5.56)	-0.87 (4.07)	-0.10 (3.25)	-0.45 (3.01)

*Note*. Anxiety, depression and externalising symptoms were measured via self-report

* *p* < .050, ** *p* < .010

^a^ – Mean difference scores reflect symptom change over 12-month period. Negative scores reflect a decrease in symptom scores and positive scores an increase.

**Table 3:** *Results of Linear Regressions for Baseline Cognitive Predictors of Concurrent Symptoms*

	Model 1^+^	Model 2^+^
	β	β
**Child report**		
Anxiety	R^2^ = 0.59, F(3, 90) = 55.67**	R^2^ = 0.12, F(3, 89) = 11.37**
Appraisals	0.59**	0.48**
Coping	0.16	0.14
Memory	0.15	0.13
Depression	R^2^ = 0.57, F(3, 90) = 55.53**	R^2^ = 0.11, F(3, 89) = 10.60**
Appraisals	0.70**	0.57**
Coping	0.06	0.03
Memory	0.09	0.07
Externalising	R^2^ = 0.30, F(3, 88) = 12.74**	R^2^ = 0.04, F(3, 87) = 1.58
Appraisals	0.60**	0.38*
Coping	− 0.08	− 0.12
Memory	0.07	0.02
**Carer-report**		
Externalising	R^2^ = 0.11, F(2, 82) = 5.49**	R^2^ = 0.01, F(2, 81) = 0.27
Appraisals	0.28	0.09
Coping	0.10	0.06
Internalising	R^2^ = 0.15, F(3, 81) = 4.82**	R^2^ = 0.02, F(3, 80) = 0.62
Appraisals	0.31	0.20
Coping	0.07	0.05
Memory	0.08	0.05

*Note.*^+^Model 1 controlled for age and sex for all outcomes, and additionally maltreatment severity for carer-reported outcomes. Model 2 also controlled for baseline PTSS.

* *p* < .050, ** *p* < .010

In the longitudinal analyses, changes in the combined cognitive processes significantly predicted change in anxiety symptoms over the 12-months, accounting for 41% of variance. Both negative appraisals (β = 0.24, *p* = .014) and maladaptive coping strategies (β = 0.33, *p* < .001) uniquely predicted change in anxiety symptoms. When additionally controlling for change in PTSS, changes in cognitive processes remained a significant predictor of anxiety, accounting for 22% of variance, with negative appraisals (β = 0.22, *p* = .029) and maladaptive coping strategies (β = 0.29, *p* = .002) remaining unique predictors (Table 4).

**Table 4:** *Results of Linear Regression Analyses Examining Change in Cognitive Processes as a Predictor of Symptom Change*

	Model 1^+^		Model 2^+^	
	β	β		
**Child report**				
Anxiety	R^2^ = 0.41, F(3, 67) = 42.57**		R^2^ = 0.22, F(3, 66) = 23.71**	
Appraisals	0.24*		0.22*	
Coping	0.33**		0.29**	
Memory	0.17		0.16	
Depression	R^2^ = 0.27, F(3, 67) = 18.90**		R^2^ = 0.14, F(3, 66) = 9.71**	
Appraisals	0.10		0.07	
Coping	0.33**		0.29*	
Memory	0.18		0.17	
Externalising	R^2^ = 0.17, F(3, 65) = 8.50**	R^2^ = 0.18, F(3, 64) = 12.18**		
Appraisals	− 0.05	0.12		
Coping	0.49**	0.34**		
Memory	− 0.01	0.16		
**Carer-report**				
Externalising	R^2^ = 0.02, F(1, 57) = 3.56		R^2^ = 0.003, F(1, 56) = 0.43	
Coping	0.18		0.07	

*Note.*^+^Model 1 controlled for age, sex and baseline symptoms for all outcomes, and additionally maltreatment severity for carer-reported outcomes. Model 2 also controlled for a change in PTSS.

* *p* < .05, ** *p* < .01

### Depression

As with anxiety, at baseline each cognitive process was significantly associated with depression symptoms (Table 1), and changes in each process over 12-months were associated with changes in depression symptoms (Table 2). After controlling for age and sex, each baseline cognitive process (appraisals, memory, coping) remained significantly positively associated with concurrent depression, explaining 57% of variance in symptoms. As with anxiety, negative appraisals were the sole unique predictor of depression symptoms (β = 0.70, *p* < .001). When additionally controlling for baseline PTSS, the combined cognitive processes remained a significant predictor, accounting for 11% of variance, with negative appraisals remaining the only unique predictor of concurrent depression severity (β = 0.57, *p* < .001).

In the longitudinal analyses, after controlling for age, sex, and baseline depression symptoms, changes in the cognitive processes significantly predicted 12-month depression symptoms, accounting for 27% of variance. Maladaptive coping strategies uniquely predicted depression symptom change (β = 0.33, *p* = .003). When additionally controlling for change in PTSS, combined cognitive processes continued to account for 14% of variance and maladaptive coping strategies remained a unique predictor (β = 0.29, *p* = .012). However, this finding was not robust to multiple imputation. Here, the combined cognitive processes continued to predict 12-month depression symptoms, but maladaptive coping was no longer a unique predictor (β = 0.26, p = .094).

### Externalising

At baseline, each cognitive process was associated with concurrent self-reported externalising symptoms, although at 12-months, this association was only present for appraisals and memory quality (Table 1). Changes in each process were associated with changes in externalising symptoms over 12-months (Table 2). Regression analysis, controlling for age and sex, showed that, at baseline, the three combined cognitive processes accounted for 30% of variance in symptoms, with negative appraisals the only unique predictor (β = 0.60, *p* < .001). When additionally controlling for baseline PTSS, the three combined processes were no longer associated with externalising symptoms, however appraisals remained a unique predictor (β = 0.38, *p* < .036).

In the longitudinal analyses, changes in the three cognitive processes predicted externalising symptoms at 12-months, accounting for 17% of variance. Maladaptive coping strategies uniquely predicted a change in externalising symptoms (β = 0.49, *p* < .001). When controlling for a change in PTSS, in addition to age, sex and baseline externalising symptoms, cognitive processes continued to account for 18% of variance, and coping remained a unique predictor (β = 0.34, *p* = .005).

### Carer-Reported Internalising and Externalising Symptoms

#### Internalising Symptoms

After controlling for age, sex, and maltreatment severity, the combined baseline cognitive processes accounted for 15% of variance in carer-reported internalising scores, however no single cognitive process was a unique predictor. After additionally controlling for PTSS, the combined cognitive processes were no longer significantly associated with baseline carer-reported internalising symptoms. As no changes in the cognitive processes were associated with a change in carer-reported internalising symptoms over 12-months (Table 2), no longitudinal analyses were run.

#### Externalising Symptoms

After controlling for age, sex, and maltreatment severity, baseline negative appraisals and maladaptive coping combined accounted for 11% of variance in externalising symptoms, however neither cognitive process was a unique predictor. When additionally controlling for PTSS, these two cognitive processes no longer explained significant variance in concurrent externalising symptoms. As only changes in maladaptive coping strategies were associated with a change in externalising symptoms (Table 2), only this process was included in the longitudinal regression models. After controlling for age, sex, maltreatment severity, and baseline externalising scores, changes in maladaptive coping did not significantly predict changes in carer-reported externalising symptoms.

## Discussion

The aim of this study was to explore whether trauma-related cognitive processes were associated with broader psychological outcomes in a sample of young people in out-of-home care. Here, we specifically focused on anxiety, depression, and externalising difficulties. We found that negative appraisals were cross-sectionally associated with self-reported anxiety, depression, and externalising symptoms, even when controlling for co-occurring PTSD symptoms. Longitudinally, negative appraisals and maladaptive coping predicted self-reported anxiety symptoms, while maladaptive coping predicted self-reported depression and externalising symptoms. These findings remained after controlling for PTSD symptoms. Findings were not replicated when using carer-reported symptoms.

These findings add to the limited evidence for the role of posttrauma cognitive processes in relation to broader psychopathology in young people exposed to complex trauma (de Haan et al., 2017; Leeson & Nixon, 2011), as well as to the limited evidence on mechanisms driving mental health difficulties in young people in out-of-home care (Hiller et al., 2021; Tarren-Sweeney, 2008). We replicated findings from both acute and multiple trauma samples that negative appraisals are associated with concurrent anxiety and depression (de Haan et al., 2017, Leeson & Nixon, 2011; Liu & Chen, 2015), and also found appraisals to predict longer-term self-reported anxiety, even after controlling for PTSD symptoms. This finding fits with existing theoretical perspectives of anxiety and depression and the central role that appraising information about the world or self in an overly negative or threatening manner plays in driving symptoms (Lau & Waters, 2017). It also provides evidence that mechanisms which are associated with posttrauma anxiety and depression in general samples of trauma exposed children are relevant for young people in out-of-home care. Whilst we found that changes in negative appraisals were associated with a change in depression severity, the role of negative appraisals in relation to depression was not robust to longitudinal regression analyses, suggesting that while the two are associated, appraisals are not necessarily driving depression symptoms in this sample.

For depression, and to a lesser extent anxiety, maladaptive coping was a unique predictor of concurrent and longer-term symptoms. These findings are consistent both with previous research on acute trauma exposed young people (e.g., Hiller et al., 2019), and with existing theoretical models of non-trauma specific depression and anxiety, in which coping strategies such as rumination and thought suppression are widely implicated as both risk and maintenance factors (Aldao et al., 2010).

The finding that negative appraisals and maladaptive coping are associated with broader posttrauma psychopathology in young people in care has important implications for treatment. For example, there is evidence that treatment outcomes for depression, when using standard best evidenced depression treatments, are less favourable for young people with a history of childhood maltreatment (Nanni et al., 2012). That is, standard treatments are less effective where a young person has a history of maltreatment. At present, trauma-focused cognitive behaviour therapies (tf-CBTs) are recommended for young people presenting with PTSD or clinically elevated symptoms of PTSS (NICE, 2018), and our findings indicate that aspects of this treatment approach are also likely to target mechanisms which drive symptoms of anxiety and depression for young people in care. Tf-CBTs include a focus on “restructuring trauma-related meanings” (NICE, 2018), which is likely to impact on trauma related negative appraisals serving to maintain symptoms of anxiety. Another core element of tf-CBTs is addressing coping strategies, including learning to overcome avoidance (Cohen & Mannarino, 2008), a key maladaptive coping mechanism which contributed to anxiety and depression symptoms in this sample. There is existing evidence that changes in trauma-related negative appraisals mediate treatment effects for depression (Jensen et al., 2018; Knutsen et al., 2018) and that the exposure element of tf-CBTs can moderate outcomes relating to anxiety and fear of talking or thinking about the trauma (Deblinger et al., 2011). For young people exposed to complex trauma, it may be that tf-CBTs could provide a more useful intervention for broader trauma-related internalising difficulties (even in the absence of PTSD), than standard CBT for anxiety or depression. This remains an ongoing important avenue for future research.

We also found evidence of an association between increased maladaptive trauma-specific cognitions and self-reported externalising difficulties. While all three cognitive processes were correlated with baseline self-reported externalising difficulties, appraisals were the only unique predictor in the cross-sectional regression model. This finding replicates associations found in samples of young people exposed to acute trauma, using parent report of symptoms (Hiller et al., 2019; Liu & Chen, 2015), and those exposed to complex trauma reporting on their own symptoms (de Haan et al., 2017). In our longitudinal models, maladaptive appraisals were no longer a unique driver of externalising difficulties. However, maladaptive coping (e.g., thought suppression) was a unique predictor of externalising symptoms at 12-months, even after controlling for PTSD symptoms. At present, the relationship between these two variables has been largely unexplored in the literature. Further research is required to understand the drivers of externalising difficulties in young people exposed to complex trauma and consideration must be given to who reports on symptoms. While parent report is deemed to be the most accurate method of assessment for externalising difficulties (Smith, 2007), for young people in out-of-home care it may be that their caregiver has not known them for an extended period of time. Consequently, for this population of young people it is not always clear who is best placed to report on symptoms of this nature.

While more disorganised or fragmented trauma memories were associated with greater internalising and externalising difficulties, they were not a unique predictor of symptoms over and above the other cognitive processes. There is evidence from the broader trauma field that trauma memory qualities (i.e., disorganised, sensory-laden, or fragmented memories) contribute to PTSD severity in children exposed to acute trauma (McGuire et al., 2021; McKinnon et al., 2017), however this has not been robustly replicated in the limited research on those exposed to more complex trauma (Wiseman et al., 2019). In this sample, we found trauma memory quality to be a less relevant mechanism for internalising and externalising difficulties. This finding is also consistent with that reported by Hiller et al. (2021) in which memory quality was also not a unique predictor of PTSD symptoms in the same sample. While there is some evidence that maltreatment may disrupt how memories are typically encoded (e.g., McCrory & Viding, 2015), the literature is mixed as to whether this is a predictor of psychopathology (Hiller et al., 2021; Ogle et al., 2013). Further research is required in order to better understand the association between trauma memories and mental health difficulties in young people exposed to complex trauma.

This study has a number of key strengths, including the longitudinal design and focus on an under-researched group of vulnerable young people who have been exposed to complex trauma, however the findings must be considered in light of its limitations. First, only young people reported on cognitive processes, which introduces single-informant bias. Indeed, when using carer-reported internalising and externalising difficulties as the outcome, we did not replicate the associations found based on young person reports. However, our focus on self-report for internal processes and symptoms followed guidance on best practice (Meiser-Stedman et al., 2007). Furthermore, with this specific population, it is often the case that carers may not have known the young person for an extended period of time, making it more difficult for them to report on mental health symptoms. Second, although no significant differences were found on baseline measures between those who completed the follow-up and those who did not, there may be other factors which were not measured which led to non-completion. However, our findings were robust to multiple imputation for missing data. Third, as social workers provided consent for young people to take part in the study (as is standard practice in research of this nature), we cannot rule out potential selection bias, although the demographics of our sample were broadly in line with national statistics for young people in out-of-home care. Fourth, as we did not collect information regarding access to mental health support between the two assessment timepoints, we were unable to examine any possible impact of this on symptom change. However, overall, we found no significant change in either mental health outcomes or cognitive processes over the 12-month period, with the exception of maladaptive coping strategies. While this may also have limited our ability to detect predictive effects, this lack of change is reflective of the complex needs of the sample in which mental health difficulties are often maintained over time (Hiller et al., 2022; Tarren-Sweeney & Goemans, 2019). Lastly, it is recognised that there is some overlap between a small number of items on the negative appraisal and depression measures. Whilst this does create the potential for slightly inflated correlations, all items were retained within the analyses in keeping with the wider literature, where similarly high levels of correlation between these two measures are reported (e.g., de Haan et al., 2017, 2019; Hiller et al., 2019; Leeson & Nixon, 2011; Liu & Chen, 2012). Also of note, the depression measure used was a well validated measure of standard depression symptoms, whilst the appraisals measure was trauma specific.

To conclude, our findings provide evidence for the applicability of trauma-related cognitive processes to broader mental health difficulties for young people in out-of-home care. While their mental health needs may be more complex than other young people, we found relatively robust evidence that the trauma-specific mechanisms that underlie internalising and externalising difficulties are the same as in other samples of trauma-exposed young people. Both maladaptive coping strategies and trauma-related negative appraisals were found to play a role in the development and maintenance of anxiety, depression and self-reported externalising symptoms, even when accounting for co-occurring PTSD symptoms. Our findings indicate that these specific posttrauma cognitive processes are likely to be important treatment targets for young people who have been exposed to complex trauma, including young people in care, who present to mental health services with elevated symptoms of anxiety and depression, regardless of the presence of PTSD symptoms.

## References

[CR1] Aldao A, Nolen-Hoeksema S, Schweizer S (2010). Emotion-regulation strategies across psychopathology: a meta-analytic review. Clinical Psychology Review.

[CR2] Allwood MA, Esposito-Smythers C, Swenson LP, Spirito A (2014). Negative cognitions as a moderator in the relationship between PTSD and substance use in a psychiatrically hospitalized adolescent sample. Journal of Traumatic Stress.

[CR4] Ashley, C., & Roth, D. (2015). What happens to siblings in the care system? Family Rights Group. https://www.standupforsiblings.co.uk/wp-content/uploads/2018/03/siblings-in-care-final-report-january-2015.pdf

[CR7] Bronsard, G., Alessandrini, M., Fond, G., Loundou, A., Auquier, P., Tordjman, S., & Boyer, L. (2016). The prevalence of mental disorders among children and adolescents in the child welfare system: a systematic review and meta-analysis. *Medicine*, *95*(7), 10.1097/MD.0000000000002622. 10.1097/MD.0000000000002622 PMC4998603 26886603

[CR8] Carr CP, Martins CMS, Stingel AM, Lemgruber VB, Juruena MF (2013). The role of early life stress in adult psychiatric disorders: a systematic review according to childhood trauma subtypes. The Journal of Nervous and Mental Disease.

[CR9] Cohen JA, Mannarino AP (2008). Trauma-focused cognitive behavioural therapy for children and parents. Child and Adolescent Mental Health.

[CR10] de Haan A, Ganser HG, Münzer A, Witt A, Goldbeck L (2017). Dysfunctional maltreatment-related cognitions in children and adolescents. Child and Adolescent Psychiatry and Mental Health.

[CR11] de Haan A, Tutus D, Goldbeck L, Rosner R, Landolt MA (2019). Do dysfunctional posttraumatic cognitions play a mediating role in trauma adjustment? Findings from interpersonal and accidental trauma samples of children and adolescents. European Journal of Psychotraumatology.

[CR12] de Haan A, Landolt MA, Fried EI, Kleinke K, Alisic E, Bryant R, Meiser-Stedman R (2020). Dysfunctional posttraumatic cognitions, posttraumatic stress and depression in children and adolescents exposed to trauma: a network analysis. Journal of Child Psychology and Psychiatry.

[CR13] Deblinger E, Mannarino AP, Cohen JA, Runyon MK, Steer RA (2011). Trauma-focused cognitive behavioral therapy for children: impact of the trauma narrative and treatment length. Depression and Anxiety.

[CR14] Department for Education (2021). *Children looked after in England including adoptions*.

[CR15] Ebesutani C, Korathu-Larson P, Nakamura BJ, Higa-McMillan C, Chorpita B (2017). The revised child anxiety and depression scale 25–parent version: scale development and validation in a school-based and clinical sample. Assessment.

[CR16] Ehlers A, Clark DM (2000). A cognitive model of posttraumatic stress disorder. Behaviour Research and Therapy.

[CR18] Gómez de La Cuesta G, Schweizer S, Diehle J, Young J, Meiser-Stedman R (2019). The relationship between maladaptive appraisals and posttraumatic stress disorder: a meta-analysis. European Journal of Psychotraumatology.

[CR19] Goodman R (2001). Psychometric properties of the strengths and difficulties questionnaire. Journal of the American Academy of Child & Adolescent Psychiatry.

[CR20] Hiller RM, Creswell C, Meiser-Stedman R, Lobo S, Cowdrey F, Lyttle MD, Halligan SL (2019). A longitudinal examination of the relationship between trauma-related cognitive factors and internalising and externalising psychopathology in physically injured children. Journal of Abnormal Child Psychology.

[CR21] Hiller, R., Fraser, A., Denne, M., Bauer, A., & Halligan, S. L. (2022). The development of young peoples’ internalising and externalising difficulties over the first three-years in the public care system. *Child Maltreatment*, 10775595211070765. https://doi.org/10.1177%2F10775595211070765. 10.1177/10775595211070765 PMC9716486 35081783

[CR22] Hiller RM, Meiser-Stedman R, Elliott E, Banting R, Halligan SL (2021). A longitudinal study of cognitive predictors of (complex) post‐traumatic stress in young people in out‐of‐home care. Journal of Child Psychology and Psychiatry.

[CR23] Hiller RM, Meiser-Stedman R, Lobo S, Creswell C, Fearon P, Ehlers A, Halligan SL (2018). A longitudinal investigation of the role of parental responses in predicting children’s post‐traumatic distress. Journal of Child Psychology and Psychiatry.

[CR25] Hogarth L, Martin L, Seedat S (2019). Relationship between childhood abuse and substance misuse problems is mediated by substance use coping motives, in school attending south african adolescents. Drug and Alcohol Dependence.

[CR26] Jensen TK, Holt T, Mørup Ormhaug S, Fjermestad KW, Wentzel-Larsen T (2018). Change in post-traumatic cognitions mediates treatment effects for traumatized youth—A randomized controlled trial. Journal of Counseling Psychology.

[CR27] Kaufman J, Jones B, Stieglitz E, Vitulano L, Mannarino AP (1994). The use of multiple informants to assess children’s maltreatment experiences. Journal of Family Violence.

[CR28] Kenardy J, Smith A, Spence SH, Lilley PR, Newcombe P, Dob R, Robinson S (2007). Dissociation in children’s trauma narratives: an exploratory investigation. Journal of Anxiety Disorders.

[CR29] Knutsen ML, Czajkowski NO, Ormhaug SM (2018). Changes in posttraumatic stress symptoms, cognitions, and depression during treatment of traumatized youth. Behaviour Research and Therapy.

[CR30] Lau JY, Waters AM (2017). Annual Research Review: an expanded account of information-processing mechanisms in risk for child and adolescent anxiety and depression. Journal of Child Psychology and Psychiatry.

[CR31] Leeson FJ, Nixon RD (2011). The role of children’s appraisals on adjustment following psychological maltreatment: a pilot study. Journal of Abnormal Child Psychology.

[CR32] Lewis SJ, Arseneault L, Caspi A, Fisher HL, Matthews T, Moffitt TE, Danese A (2019). The epidemiology of trauma and post-traumatic stress disorder in a representative cohort of young people in England and Wales. The Lancet Psychiatry.

[CR33] Lewis S, Koenen K, Ambler A, Arseneault L, Caspi A, Fisher H, Danese A (2021). Unravelling the contribution of complex trauma to psychopathology and cognitive deficits: a cohort study. The British Journal of Psychiatry.

[CR34] Liu ST, Chen SH (2015). A community study on the relationship of posttraumatic cognitions to internalizing and externalizing psychopathology in taiwanese children and adolescents. Journal of Abnormal Child Psychology.

[CR35] McCrory EJ, Viding E (2015). The theory of latent vulnerability: reconceptualizing the link between childhood maltreatment and psychiatric disorder. Development and psychopathology.

[CR36] McGuire R, Hiller RM, Ehlers A, Fearon P, Meiser-Stedman R, Leuteritz S, Halligan SL (2021). A longitudinal investigation of children’s trauma memory characteristics and their relationship with posttraumatic stress disorder symptoms. Research on Child and Adolescent Psychopathology.

[CR37] McKinnon A, Brewer N, Meiser-Stedman R, Nixon RDV (2017). Trauma memory characteristics and the development of acute stress disorder and post-traumatic stress disorder in youth. Journal of Behavior Therapy and Experimental Psychiatry.

[CR38] Meiser-Stedman R, Smith P, Bryant R, Salmon K, Yule W, Dalgleish T, Nixon RDV (2009). Development and validation of the child post-traumatic Cognitions Inventory (CPTCI). Journal of Child Psychology and Psychiatry.

[CR39] Meiser-Stedman R, Smith P, Glucksman E, Yule W, Dalgleish T (2007). Parent and child agreement for acute stress disorder, post-traumatic stress disorder and other psychopathology in a prospective study of children and adolescents exposed to single-event trauma. Journal of Abnormal Child Psychology.

[CR41] Mitchell R, Brennan K, Curran D, Hanna D, Dyer KF (2017). A meta-analysis of the association between appraisals of trauma and posttraumatic stress in children and adolescents. Journal of Traumatic Stress.

[CR42] Nanni V, Uher R, Danese A (2012). Childhood maltreatment predicts unfavorable course of illness and treatment outcome in depression: a meta-analysis. American Journal of Psychiatry.

[CR43] National Institute of Health and Care Excellence (2018). Post-traumatic stress disorder (NG116). https://www.nice.org.uk/guidance/ng116 31211536

[CR44] National Institute of Health and Care Excellence (2019). Antisocial behaviour and conduct disorders in children and young people: recognition and management (CG158). https://www.nice.org.uk/guidance/cg158 32073810

[CR45] O’Kearney R, Speyer J, Kenardy J (2007). Children’s narrative memory for accidents and their post-traumatic distress. Applied Cognitive Psychology: The Official Journal of the Society for Applied Research in Memory and Cognition.

[CR46] Ogle CM, Block SD, Harris LS, Goodman GS, Pineda A, Timmer S, Saywitz KJ (2013). Autobiographical memory specificity in child sexual abuse victims. Development and Psychopathology.

[CR47] Parliament, House of Lords. (2012). Debate on 25 October: Looked-after-children. Library Note: LLN 2012/036. https://researchbriefings.files.parliament.uk/documents/LLN-2012-036/LLN-2012-036.pdf

[CR48] Sachser C, Berliner L, Holt T, Jensen TK, Jungbluth N, Risch E, Goldbeck L (2017). International development and psychometric properties of the child and adolescent trauma screen (CATS). Journal of Affective Disorders.

[CR49] Salmond CH, Meiser-Stedman R, Glucksman E, Thompson P, Dalgleish T, Smith P (2011). The nature of trauma memories in acute stress disorder in children and adolescents. Journal of Child Psychology and Psychiatry.

[CR50] Shin SH, Jiskrova GK, Yoon SH, Kobulsky JM (2020). Childhood maltreatment, motives to drink and alcohol-related problems in young adulthood. Child Abuse & Neglect.

[CR51] Simkiss DE, Stallard N, Thorogood M (2012). A systematic literature review of the risk factors associated with children entering public care. Child: Care Health and Development.

[CR52] Smith SR (2007). Making sense of multiple informants in child and adolescent psychopathology: a guide for clinicians. Journal of Psychoeducational Assessment.

[CR53] Tarren-Sweeney M (2008). Retrospective and concurrent predictors of the mental health of children in care. Children and Youth Services Review.

[CR54] Tarren-Sweeney M, Goemans A (2019). A narrative review of stability and change in the mental health of children who grow up in family-based out-of-home care. Developmental Child Welfare.

[CR55] Trickey D, Siddaway AP, Meiser-Stedman R, Serpell L, Field AP (2012). A meta-analysis of risk factors for posttraumatic stress disorder in children and adolescents. Clinical Psychology Review.

[CR56] Wiseman H, Hamilton-Giachritsis C, Hiller RM (2019). The relevance of cognitive behavioral models of post-traumatic stress following child maltreatment: a systematic review. Trauma Violence & Abuse.

